# Determinants of Recovery from Severe Posterior Reversible Encephalopathy Syndrome

**DOI:** 10.1371/journal.pone.0044534

**Published:** 2012-09-14

**Authors:** Stephane Legriel, Olivier Schraub, Elie Azoulay, Philippe Hantson, Eric Magalhaes, Isaline Coquet, Cedric Bretonniere, Olivier Gilhodes, Nadia Anguel, Bruno Megarbane, Laurent Benayoun, David Schnell, Gaetan Plantefeve, Julien Charpentier, Laurent Argaud, Bruno Mourvillier, Arnaud Galbois, Ludivine Chalumeau-Lemoine, Michel Rivoal, François Durand, Arnaud Geffroy, Marc Simon, Annabelle Stoclin, Jean-Louis Pallot, Charlotte Arbelot, Martine Nyunga, Olivier Lesieur, Gilles Troché, Fabrice Bruneel, Yves-Sébastien Cordoliani, Jean-Pierre Bedos, Fernando Pico

**Affiliations:** 1 Medical-Surgical Intensive Care Department, CH Versailles – Site André Mignot, Le Chesnay, France; 2 Medical Intensive Care Department, CHU Saint-Louis, Paris, France; 3 Intensive Care Department, Cliniques Universitaires Saint-Luc, Brussels, Belgium; 4 Neurology Department and Stroke Center, CH Versailles - André Mignot, Le Chesnay, France; 5 Medical-Surgical Intensive Care Department, Hopital Foch, Suresnes, France; 6 Intensive Care Department, CHU de Nantes - Hôtel Dieu, Nantes, France; 7 Medical-Surgical Intensive Care Department, CHI de Créteil, Créteil, France; 8 Medical Intensive Care Department, Chu de Bicêtre, Kremlin-Bicêtre, France; 9 Medical Intensive Care Department, CHU Lariboisière, Paris, France; 10 Surgical Intensive Care Department, CHU Beaujon, Clichy, France; 11 Medical-Surgical Intensive Care Department, CH Victor Dupouy, Argenteuil, France; 12 Medical Intensive Care Department, CHU Cochin, Paris, France; 13 Medical Intensive Care, Hospices Civils de Lyon, Hôpital Edouard Herriot, Lyon, France; 14 Medical Intensive Care Department, CHU Bichat, Paris, France; 15 Medical Intensive Care Department, CHU Saint Antoine, Paris, France; 16 Medical Intensive Care Department, CHU Tenon, Paris, France; 17 Medical-Surgical Intensive Care Department, CH Arpajon, Arpajon, France; 18 Hepatology and Liver Intensive care, CHU Beaujon, Clichy, France; 19 Surgical Intensive Care Department, CHU Bichat, Paris, France; 20 Intensive Care Department, Cliniques du Sud-Luxembourg of Arlon, Arlon, Belgium; 21 Medical-Surgical Intensive Care Department, Institut Gustave Roussy, Villejuif, France; 22 Medical-Surgical Intensive Care Department, CH André Grégoire, Montreuil, France; 23 Surgical Intensive Care Department, CHU Pitié Salpétrière, Paris, France; 24 Medical-Surgical Intensive Care Department, CH de Roubaix, Roubaix, France; 25 Medical-Surgical Intensive Care Department, CH de La Rochelle, La Rochelle, France; 26 Radiology Department, Hôpital privé de Parly II, Le Chesnay, France; Università Vita-Salute San Raffaele, Italy

## Abstract

**Objective:**

Few outcome data are available about posterior reversible encephalopathy syndrome (PRES). We studied 90-day functional outcomes and their determinants in patients with severe PRES.

**Design:**

70 patients with severe PRES admitted to 24 ICUs in 2001–2010 were included in a retrospective cohort study. The main outcome measure was a Glasgow Outcome Scale (GOS) of 5 (good recovery) on day 90.

**Main Results:**

Consciousness impairment was the most common clinical sign, occurring in 66 (94%) patients. Clinical seizures occurred in 57 (81%) patients. Median mean arterial pressure was 122 (105–143) mmHg on scene. Cerebral imaging abnormalities were bilateral (93%) and predominated in the parietal (93%) and occipital (86%) white matter. Median number of brain areas involved was 4 (3–5). Imaging abnormalities resolved in 43 (88%) patients. Ischaemic and/or haemorrhagic complications occurred in 7 (14%) patients. The most common causes were drug toxicity (44%) and hypertensive encephalopathy (41%). On day 90, 11 (16%) patients had died, 26 (37%) had marked functional impairments (GOS, 2 to 4), and 33 (56%) had a good recovery (GOS, 5). Factors independently associated with GOS<5 were highest glycaemia on day 1 (OR, 1.22; 95%CI, 1.02–1.45, *p* = 0.03) and time to causative-factor control (OR, 3.3; 95%CI, 1.04–10.46, *p* = 0.04), whereas GOS = 5 was associated with toxaemia of pregnancy (preeclampsia/eclampsia) (OR, 0.06; 95%CI, 0.01–0.38, *p* = 0.003).

**Conclusions:**

By day 90 after admission for severe PRES, 44% of survivors had severe functional impairments. Highest glycaemia on day 1 and time to causative-factor control were strong early predictors of outcomes, suggesting areas for improvement.

## Introduction

Posterior reversible encephalopathy syndrome (PRES) is a clinicoradiologic entity characterized by a variable combination of consciousness impairment, seizure activity, headaches, visual abnormalities, nausea/vomiting, and focal neurological signs. [1], [2], [3], [4], [5] Cerebral imaging abnormalities are often symmetric and predominate in the posterior white matter. Oedema is an occasional finding in the frontal and temporal lobes, basal ganglia, cerebellum, brainstem, and cortical grey matter. [4], [5], [6] Recognition of PRES is improving with the increasing availability of magnetic resonance imaging (MRI) and recent reappraisal of the imaging abnormality spectrum. [6].

The pathophysiology of PRES remains controversial, and the two main hypotheses contradict each other. One involves impaired cerebral autoregulation responsible for an increase in cerebral blood flow, whereas the other incriminates endothelial dysfunction with cerebral hypoperfusion. This hypoperfusion hypothesis may be most relevant to cases of PRES associated with cytotoxic therapy. Under both hypotheses, the cerebral blood perfusion abnormalities result in blood-brain barrier dysfunction with cerebral vasogenic oedema. [4], [5], [7] PRES can develop in association with a vast array of clinical conditions and is typically reversible once the cause is removed. [3], [8], [9] However, its reversible nature has been challenged based on reports of permanent neurological impairments and of mortality rates reaching 15% [8], [9]. No studies focusing specifically on patients with severe PRES requiring life-sustaining treatments [10], [11], [12], [13] have been published to date. Although the pathophysiological mechanisms may be the same as in less severe forms, knowledge of factors influencing the outcome of severe PRES might result in improved early management. [7].

Here, our objective was to identify predictors of functional outcome on day 90 in adults with severe PRES, with special attention to factors amenable to improvement. Thus, we thought to provide intensivists and other clinicians a realistic picture of severe PRES with useful management pathways in daily clinical practice. To this end, we conducted a multicentre retrospective cohort study.

## Methods

The ethics committee of the French Society for Critical Care approved the constitution of this retrospective cohort of patients with severe PRES.

### Patients

Patients admitted to one of the 24 participating ICUs (Appendix) between May 2001 and May 2010 and exhibiting clinical and neuroimaging features consistent with severe PRES, as defined below, were eligible for the study.

First selection of cases was performed by searching a patient’s hospital claims data for the presence of certain International Statistical Classification of Diseases and Related Health Problems 10th Revision diagnosis and procedure codes among : Encephalopathy (G93.4), Hypertensive encephalopathy (I67.4), Toxic encephalopathy (G92), Gestational Hypertension (O14), Eclampsia (O15), Unspecified maternal hypertension (O16), Convulsions (R56), Epilepsy (G40), Status epilepticus (G41), Headache (G44), Visual disturbances (H53 and H54), Cerebral oedema (G93.6) and Abnormal findings on diagnostic imaging and in function studies, without diagnosis (R90–R94).

All neuroimaging documents were reviewed by two independent and certified neurologists with specialist stroke-experience and trained in MRI diagnosis of PRES and its pitfalls. Neuroimaging review was performed blinded to the clinical findings. Patients were included by consensus between two neurologists (FP and EM). Patients with no brain imaging studies available for review by the study neurologists were not included.

### Definitions

PRES was defined as a variable combination of acute neurologic clinical changes including consciousness impairment, seizure activity, headaches, visual abnormalities, nausea/vomiting, and focal neurological signs [4], [5] associated with neuroimaging findings consistent with PRES [4], including vasogenic oedema by MRI diffusion sequences (measurement of apparent diffusion coefficient (ADC)) or at least partial reversibility on follow-up imaging when diffusion sequences were not available. [6].

PRES was severe when associated with neurological failure defined by any neurological disorder of central origin among impairment of consciousness, seizure with or without status epilepticus, focal sign, encephalopathy, and meningeal symptoms, which required intensive care management for monitoring or life support management.

Coma was defined as the absence of arousal and consciousness with a Glasgow Coma Scale (GCS) score <9. [14], [15].

Visual abnormalities consisted of blurred vision, visual neglect, homonymous hemianopsia, visual hallucinations, and cortical blindness.

Focal neurological signs consisted of symptoms or signs related with damage to, or dysfunction of, a specific anatomic site in the central nervous system. [16] These signs were categorized as unifocal or multifocal, and as transient or persistent.

Convulsive status epilepticus was defined as continuous motor seizure activity for at least 5 minutes (continuous) or as more than two motor seizures without full recovery of consciousness in the interval (intermittent). [17], [18] Refractory status epilepticus was defined as continuous or intermittent seizures despite treatment with an intravenous benzodiazepine (clonazepam or diazepam) and intravenous phenytoin, fosphenytoin, or phenobarbital. [19] Electrical status epilepticus was diagnosed in comatose patients with or without subtle convulsive movements (rhythmic twitching of the arms, legs, trunk, or facial muscles; tonic eye deviation; or nystagmoid eye jerking) [20] but with generalized ictal discharges on the electroencephalogram (EEG). [21].

Seizure activity on the EEG was defined as continuous or recurrent rhythmic focal or generalized spikes; sharp waves; spike waves; or rhythmic waves changing in amplitude, frequency, and/or spatial distribution. [22].

Hypertension was defined according to the 2007 European guidelines for the management of arterial hypertension. Grade 1: mild hypertension (systolic blood pressure [SBP], 140–159 mmHg and/or diastolic blood pressure [DBP], 90–99 mmHg); Grade 2: moderate hypertension (SBP, 160–179 mmHg and/or DBP 100–109 mmHg); and Grade 3: severe hypertension (SBP≥180 mmHg and/or DBP≥110 mmHg). [23].

### Diagnosis and Treatment

The management combined symptomatic life-supporting treatments and control of the factor causing PRES. [10], [11] Efforts were made to control systemic secondary brain insults and to limit effects of potential cranial hypertension Hypoglycemia was routinely checked and corrected. If glucose was given, 100 mg of thiamine was administered concomitantly, most notably when there was evidence of vitamin B1 deficiency. Patients were also routinely evaluated for hyperthermia, hyperglycemia, hypo- or hyper-carbia, anemia, metabolic disturbances, epileptic activity and aspiration pneumonia that may complicate the initial consciousness disorders and which required prompt correction. Patients with status epilepticus were managed as previously described. [24] Control of severe hypertension, if present, was an important part of the symptomatic management. Intravenous antihypertensive drugs including labetolol, nicardipine, or urapidil were given. [25].

A neurologist consultant was available for advice. Extensive diagnostic investigations were performed. CT was easier to obtain first. MRI was performed in most patients, as either the first or the second imaging study. Both local radiologists and neurologist consultants read the neuroimaging studies. In addition, a neuroradiologist was consulted if deemed necessary. Electroencephalography (EEG) was performed routinely to look for non-convulsive status epilepticus. Cerebrospinal fluid was examined in patients with a fever or clinical suspicion of meningitis and when deemed appropriate by the attending physicians. Laboratory tests were also obtained routinely. Plasma anticonvulsant drug assays, including magnesium assays, and qualitative tests for toxic agents or medications associated with seizures and other symptoms of PRES were performed at the discretion of the attending physicians.

Interventions to control identified causative factors were initiated promptly. These interventions included blood pressure control, withdrawal of cancer chemotherapy or immunosuppressive agents, caesarean section, and/or dialysis, as appropriate.

### Data Collection

**Figure 1:**
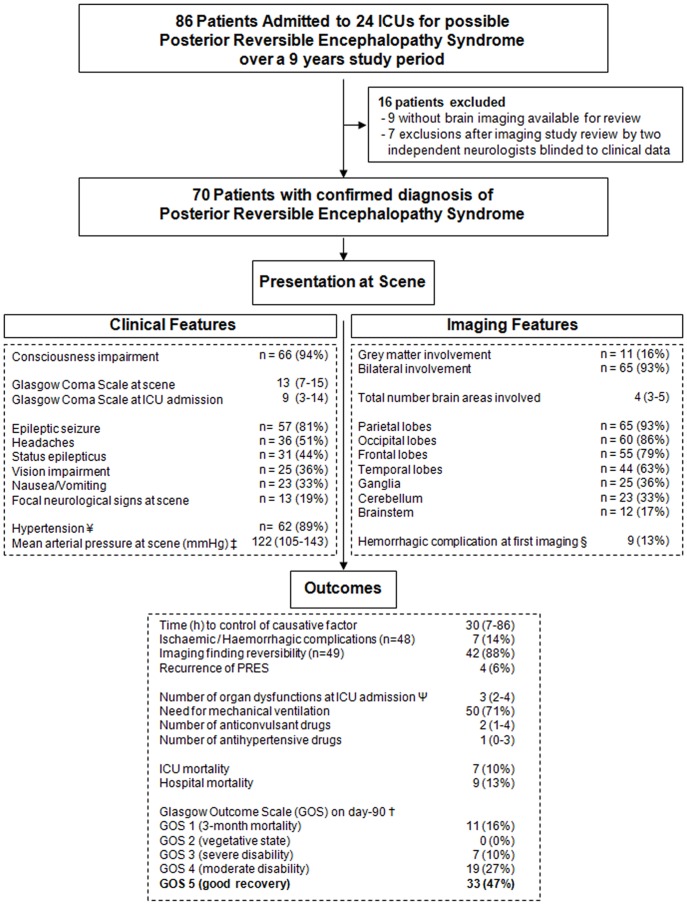
Patient flow chart, clinicoradiologic features, management, and 90-day follow-up in 70 patients with severe posterior reversible encephalopathy syndrome. ¥ Hypertension was defined according to the 2007 European guidelines for the management of arterial hypertension(16). Grade 1: mild hypertension (systolic blood pressure [SBP], 140–159 mmHg and/or diastolic blood pressure [DBP], 90–99 mmHg); Grade 2: moderate hypertension (SBP, 160–179 mmHg and/or DBP, 100–109 mmHg); Grade 3: severe hypertension (SBP≥180 mmHg and/or DBP≥110 mmHg) ‡ Mean arterial pressure (2/3 diastolic +1/3 systolic pressure) § Nine patients with haemorrhagic complications at first imaging: 3 with no follow-up imaging studies, 3 with persistent haemorrhagic abnormalities by follow-up imaging, and 3 with resolution of the haemorrhagic abnormalities Ψ According to the Logistic Organ Dysfunction (LOD) score [27] †The primary outcome measure was the score on the Glasgow Outcome Scale [39] (GOS) 90 days after onset of severe posterior reversible encephalopathy syndrome. A score of 1 indicates death; 2, a vegetative state (the patient is unable to interact with the environment); 3, severe disability (the patient is unable to live independently but can follow commands); 4, moderate disability (the patient is capable of living independently but unable to return to work or school); and 5, mild or no disability (the patient is able to return to work or school). A favourable outcome was defined as a score of 5 and an unfavourable outcome as a score lower than 5. The day-90 GOS score was known in all 70 patients.

A standardized form was used to collect the variables listed in Tables 1–7 and Figure 1. Clinical features of PRES were collected retrospectively based on data in the pre-hospital notes, emergency-room chart, and ICU chart.

**Table 1:** Categories of the structured Glasgow Outcome Scale.

Category	Classification	Description
**1**	**Death**	**Patient is certified dead.**
**2**	**Vegetative state**	**Patient is unable to interact with the environment.** *Patients who show no evidence of meaningful responsiveness. This non-sentient state must be distinguished from other conditions of wakeful, reduced responsiveness–such as the locked-in syndrome, akinetic mutism and total global aphasia. Vegetative patients breathe spontaneously, have periods of spontaneous eye-opening, may follow moving objects with their eyes, show reflex responses in their limbs (to postural or painful stimuli), and they may swallow food placed in their mouths.*
**3**	**Severe disability**	**Patient is unable to live independently but can follow commands.** *This indicates that a patient is conscious but needs the assistance of another person for some activities of daily living every day. This may range from continuous total dependency to the need for assistance with only one activity.*
**4**	**Moderate disability**	**Patient is capable of living independently but unable to return to work or school.** *Such a patient is able to look after himself at home, to get out and about to the shops and to travel by public transport. However, some previous activities, either at work or in social life, are now no longer possible by reason of either physical or mental deficit.*
**5**	**Mild or no disability**	**Patient is able to return to work or school.** *This indicates the capacity to resume normal occupational and social activities, although there may be minor physical or mental deficits. However, for various reasons, the patient may not have resumed all his previous activities, and in particular may not be working.*

(Adapted from Jennett B, Teasdale G, Braakman R, Minderhoud J, Knill-Jones R. Predicting outcome in individual patients after severe head injury. *Lancet* 1976;1∶1031–1034.)

A standardized form was used to collect the neuroimaging features of PRES: uni/bilateral involvement, grey/white matter involvement, lesion distribution (i.e., parietal and/or occipital and/or temporal and/or frontal lobes, ganglia, brainstem, cerebellum), presence of ischaemic and/or haemorrhagic complications, reversibility of lesions, and recurrences (Figure 1).

**Table 2:** Patient characteristics (n = 70).

	No. (%) or Median (IQR)
**Demographics**	
Age (y)^Ω^	36 (25–52)
Female gender	45 (64%)
Pre-existing co-morbidity	56 (80%)
**Treatments for severe PRES**	
Number of anticonvulsant drugs needed to control status epilepticus	2 (1–4)
Number of antihypertensive drugs needed to control acute hypertensive crisis	1 (0–3)
Refractory status epilepticusФ	7 (10%)
Progression to electrical status epilepticus†	10 (17%)
Need for mechanical ventilation	50 (71%)
Duration (d) of mechanical ventilation	5 (2–10)
Length (d) of hospital stay	33 (15–62)
Time (h) from PRES onset to control of causative factor	30 (7–86)
**Severity scores at ICU admission**	
SAPS II score	42 (27–53)
GCS score	9 (3–14)
**Patient characteristics at ICU admission**	
Time (d) from hospitalization to ICU admission	1 (0–12)
Time (h) from clinical acme of PRES to ICU admission	0 (-2,2–7.1)
Time (h) from PRES onset to ICU admission	0 (-2.5–0.5)
**Tests to identify cause of PRES**	
Lumbar punctureΣ	40 (57%)
CT scan and/or MRI‡	70 (100%)
Electroencephalography	59 (84.3%)
**Outcomes**	
Partial or full reversibility of imaging abnormalities (n = 49)£	43 (88%)
Ischaemic and/or haemorrhagic complication^ΨΘ^	7 (14%)
Time (d) from PRES onset to resolution of initial imaging findingsΨ	12 (7–40)
Recurrence of PRES	4 (5.7%)
Mortality rate at hospital discharge‡	9 (13%)

PRES, Posterior Reversible Encephalopathy syndrome; CT, computed tomography; MRI, magnetic resonance imaging; ICU, Intensive Care Unit; SAPS, Simplified Acute Physiology Score; [26] GCS, Glasgow Coma Scale score [14], [15].

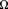
Age (yr): 38±15 (Mean ± sd).

Σ Lumbar puncture, n = 40 (57%); Average Cell count (number/µL): 17 (range, 0–320); average glucose (mmol/L): 4.1 (range, 0.6–6.6); average protein (g/L): 0.81 (range, 0.25–4.2).

‡ CT scan only, n = 12 (17%); MRI only, n = 25 (36%); CT scan and MRI, n = 33 (47%).

Ф Refractory status epilepticus was defined as continuous or intermittent seizures despite treatment with an intravenous benzodiazepine (clonazepam or diazepam) and intravenous phenytoin, fosphenytoin, or phenobarbital [19].

† Electrical status epilepticus was diagnosed when the patient was found in a coma with or without subtle convulsive movements but with generalized or lateralized ictal discharges on the electroencephalogram (n = 59). [21].

£ Reversibility was partial in 21 (43%) and total in 22 (45%) patients.

Ψ among patients with follow-up imaging studies, n = 49 (70%).

Θ haemorrhagic, n = 6 (12%); ischaemic, n = 4 (8.2%) (A given patient could have more than one complication.).

‡ Four deaths directly ascribable to PRES: one patient each had brain death related to cardiac arrest complicating the treatment of status epilepticus, refractory status epilepticus with multi-organ failure, cerebral haemorrhage with ventricular flooding, and brain death related to cerebral herniation complicating cerebral ischaemia.

The primary cause of PRES was classified as hypertensive encephalopathy, exposure to a toxic agent, toxaemia of pregnancy (preeclampsia/eclampsia), autoimmune disease, or miscellaneous causes. Toxicity was considered when PRES occurred during or after a pharmacological treatment known to induce neurological toxicity (Table 3). Hypertensive encephalopathy was diagnosed in patients presenting the association of severe hypertension and signs of neurological failure [23], and absence of evidence of other etiologic category as described above.

**Table 3:** Causes of severe posterior reversible encephalopathy syndrome (PRES) (n = 70).

Causes of PRES a	Median value of mean arterial pressure at scene (mmHg)	All patients n (%)
**Hypertensive encephalopathy**	137 (120–155)	**29 (41%)**
**Toxicity**	113 (91–127)	**31 (44%)**
Cytotoxic agents		
Cyclophosphamide		2
Cytarabine		2
Methotrexate		2
Vincristine		1
Proteasome inhibitor: Bortezomid		1
Anti-angiogenic agents: Bevacizumab		1
Monoclonal antibodies: Muromonab (anti-CD3)		1
Immunosuppressive agents		
Anticalcineurin agents		
Cyclosporine A		12
Tacrolimus (FK 506)		7
Mycophenolate mofetil		1
High-dose corticosteroid therapy		6
Other agents		
Antiretroviral agents		1
Lysergic acid amide		1
**Toxaemia of pregnancy (preeclampsia/eclampsia)**	133 (118–146)	**16 (23%)**
**Autoimmune diseases**	140 (122–157)	**8 (11%)**
Systemic lupus erythematosus		2
Systemic sclerosis (scleroderma)		1
Wegener’s granulomatosis		1
Thrombotic microangiopathy		4
**Miscellaneous causes**	126 (102–134)	**5 (7.1%)**
Hypomagnesaemia		1
Sickle cell disease		2
Infection/sepsis/shock		2

a Some patients had more than one cause.

The time from symptom onset to control of the causative factor was clocked (e.g., time to achieve blood pressure values within the normal range for hypertension, time to treatment discontinuation for toxicity, or time of C-section for toxaemia).

**Table 4:** Systemic secondary brain insults on day 1 of ICU management of patients with severe posterior reversible encephalopathy syndrome (PRES) (n = 70).

	Minimal level on day 1			Maximal level on day 1		
	All patients	GOS 1–4 on day 90	GOS 5 on day 90	All patients	GOS 1–4 on day 90	GOS 5 on day 90
Blood sodium (mmol/L)	136 (134–139)	137 (134–140)	136 (134–138)	140 (137–143)	140 (137–143)	140 (137–142)
Glycaemia (mmol/L)	5.2 (4.5–6.3)	5.4 (4.7–6.6)	5.2 (4.5–6.1)	7.9 (6.7–10.4)	8.7 (7.5–11.1)*	7.1 (6.1–9.3)*
PCO_2_ (mmHg)	30 (26–35)	30 (26–36)	30 (28–33)	36 (32–41)	37 (30–43)	35 (32–38)
Central temperature (°C)	36.5 (36–37)	36.9 (36.0–37.0)	36.4 (36.1–37.0)	38 (37.4–38.6)	38.0 (37.4–38.7)	38.0 (37.4–38.6)

* *p*<0.05.

Severity and organ dysfunction at ICU admission were assessed using the Simplified Acute Physiology Score II (SAPS-II) [26] and the Logistic Organ Dysfunction (LOD) score. [27].

**Table 5:** Patient characteristics and univariate predictors of 90-day functional outcome.

	No. (%) or Median (InterQuartile Range)			Univariate analysis		
	All Patients n = 70	GOS = 5 n = 33 (47.9%)	GOS <5 n = 37 (52.9%)	OR	95%CI	*p* value
**Demographics**						
Age (y)	36 (25–52)	34 (26–45)	41 (24–56)	1.02	0.98–1.05	0.36
Female gender	45 (64%)	23 (70%)	22 (60%)	0.64	0.24–1.72	0.37
Pre-existing co-morbidity ψ	56 (80%)	22 (67%)	34 (92%)	5.67	1.42–22.63	**0.01**
**Clinical characteristics**						
Epileptic seizure	57 (81%)	29 (88%)	28 (76%)	0.43	0.12–1.55	0.19
Status epilepticus	31 (44%)	11 (33%)	20 (54%)	2.35	0.89–6.21	0.08
Consciousness impairment	66 (94%)	30 (91%)	36 (97%)	3.60	0.56–36.43	0.28
Vision impairment	25 (36%)	11 (33%)	14 (38%)	1.22	0.45–3.25	0.69
Headache	36 (51%)	20 (61%)	16 (43%)	0.49	0.19–1.28	0.15
Nausea/Vomiting	23 (33%)	11 (33%)	12 (32%)	0.96	0.35–2.61	0.93
Focal neurological signs at scene †	13 (19%)	6 (18%)	7 (19%)	1.05	0.31–3.5	0.94
Mean arterial pressure at scene of PRES (mmHg)	122 (105–143)	121 (113–144)	125 (99–141)	0.99	0.98–1.01	0.48
**Brain imaging characteristics**						
Grey matter involvement	11 (16%)	3 (9.1%)	8 (22%)	2.76	0.66–11.43	0.16
Occipital lobes involvement	60 (86%)	30 (91%)	30 (81%)	0.43	0.10–1.82	0.25
Parietal lobes involvement	65 (93%)	31 (94%)	34 (92%)	0.73	0.11–4.69	0.74
Frontal lobes involvement	55 (79%)	27 (82%)	28 (76%)	0.69	0.22–2.21	0.53
Temporal lobes involvement	44 (63%)	21 (64%)	23 (62%)	0.94	0.35–2.48	0.89
Cerebellum involvement	23 (33%)	13 (39%)	10 (27%)	0.57	0.21–1.56	0.27
Brainstem involvement	12 (17%)	8 (24%)	4 (11%)	0.38	0.10–1.40	0.14
Ganglia involvement	25 (36%)	16 (49%)	9 (24%)	0.34	0.12–0.94	**0.04**
Total number of brain areas involved	4 (3–5)	5 (3–5)	4 (3–5)	0.71	0.51–1.01	**0.05**
Ischemic and/or hemorrhagic complication Θ	12 (17%)	3 (9.1%)	9 (24%)	7.46	0.86–64.35	**0.07**

ICU: intensive care unit; OR: odds ratio; 95% CI: 95% confidence interval; Higher scores indicate a higher risk of poor functional outcome.

ψ as indicated by a McCabe score ≥1;

† Focal neurological signs were defined as symptoms or signs consistent with damage to, or dysfunction of, a specific anatomic site in the central nervous system. Signs were unifocal or multifocal, and transient or persistent;

Θ haemorrhagic, n = 6 (12.2%); ischaemic, n = 4 (8.2%) (A given patient could have more than one complication.);

Values of *p* in bold are significant (*p*<0.05).

### Assessment of Outcome

The primary evaluation criterion was the structured Glasgow Outcome Scale (GOS) score on day 90 (±7 days) after onset of PRES (Table 1). The GOS score reflects both mortality and morbidity. [28] Each surviving patient was interviewed for GOS score determination on day 90 and day 180 by a trained physician, when allowed by the time since inclusion. Otherwise, the GOS score was extracted from the hospital charts or determined by interviewing the patient’s general practitioner or neurologist. For this study, we defined a favourable outcome as a GOS score of 5, that is, alive with good function enabling the return to former occupational or academic activities, with or without minor physical or mental deficits.

**Table 6:** Patient characteristics and univariate predictors of 90-day functional outcome.

	No. (%) or Median (InterQuartile Range)			Univariate analysis		
	All Patients n = 70	GOS = 5 n = 33 (47.9%)	GOS <5 n = 37 (52.9%)	OR	95%CI	*p* value
**Patient characteristics and severity scores at ICU admission**						
Time (h) from PRES onset to ICU admission	0 (−2.5–0.5)	0 (−2.2–18.9)	0 (−2.6–0)	1.00	0.99–1.01	0.94
SAPS II score	42 (27–53)	36 (23–53)	44 (37–55)	1.04	1.00–1.07	**0.03**
LOD score	6 (3.7–8.0)	4.5 (1.0–9.5)	6 (4.7–8.0)	1.08	0.95–1.24	0.23
GCS score	9 (3–14)	12 (4–15)	8 (3–12)	0.93	0.84–1.03	0.14
**Systemic secondary brain insults at day 1**						
Lowest blood-sodium level (mmol/l) on day 1	136 (134–139)	136 (134–138)	136 (134–140)	1.00	0.89–1.12	0.98
Highest blood-sodium level (mmol/l) on day 1	140 (137–143)	140 (137–142)	140 (138–143)	0.99	0.88–1.12	0.96
Lowest glycaemia (mmol/L) on day 1	5.2 (4.5–6.3)	5.2 (4.5–6.1)	5.3 (4.6–6.5)	1.20	0.90–1.59	0.21
Highest glycaemia (mmol/L) on day 1	7.9 (6.7–10.4)	7.1 (6.1–9.3)	8.7 (7.4–11.1)	1.17	1.01–1.37	**0.04**
Lowest PCO2 level (mmHg) on day 1	30 (26–35)	30 (28–33)	30 (26–36)	1.02	0.92–1.13	0.68
Higher PCO2 level (mmHg) on day 1	36 (32–41)	35 (32–38)	37 (29–43)	1.03	0.96–1.09	0.42
Lowest temperature level on day 1	36.5 (36–37)	36.4 (36.1–37)	36.8 (36.37)	1.34	0.73–2.57	0.32
Higher temperature level on day 1	38 (37.4–38.6)	38 (37.4–38.6)	38 (37.4–38.7)	1.08	0.69–1.70	0.74
**Treatments for severe PRES**						
Need for mechanical ventilation	50 (71%)	20 (61%)	30 (81%)	2.78	0.95–8.19	0.06
Duration (d) of mechanical ventilation	5 (2–10)	3 (2–6)	7 (4–16)	1.26	1.05–1.50	**0.01**
Refractory status epilepticusФ	7 (10%)	1 (3%)	6 (16%)	6.19	0.70–54.46	0.10
Length (d) of hospital stay	7 (4–16)	5 (3–8)	13 (6–27)	1.11	1.03–1.2	**0.008**
Length of hospital stay	33 (15–62)	18 (12–33)	60 (28–108)	1.04	1.02–1.07	**0.001**
**Cause of PRES^Y^**						
Time (h) from PRES onset to control of causative factor	30 (7–86)	17 (5–48)	40 (18–213)	1.01	1.00–1.01	**0.03**
Hypertensive encephalopathy	29 (41%)	13 (39%)	16 (43%)	1.17	0.45–1.94	0.74
Toxemia of pregnancy (preeclampsia/eclampsia)	16 (23%)	14 (42%)	2 (5.4%)	0.08	0.01–0.38	**0.002**
Toxic	31 (44%)	10 (30%)	21 (57%)	3.02	1.13–8.10	**0.03**

ICU: intensive care unit; OR: odds ratio; 95% CI: 95% confidence interval; SAPS: Simplified Acute Physiology Score; LOD: Logistic Organ Dysfunction score. Higher scores indicate a higher risk of poor functional outcome.

Ф Refractory status epilepticus was defined as continuous or intermittent seizures despite treatment with an intravenous benzodiazepine (clonazepam or diazepam) and intravenous phenytoin, fosphenytoin, or phenobarbital;

Y Some patients had more than one diagnosis; Values of *p* in bold are significant (*p*<0.05).

### Statistical Analysis

Quantitative parameters are reported as median and interquartile range (IQR, 25th–75th percentile) and qualitative parameters as numbers and percentage. Categorical variables were compared using the χ2 test or Fisher’s exact test, as appropriate. Continuous variables were compared using the Mann-Whitney U test or the Wilcoxon test, as appropriate. The day-90 GOS score was known for all study patients. Associations between patient characteristics and 90-day GOS score were assessed using a logistic regression model (Tables 5 and 6). Multivariable analysis was performed using stepwise forward selection to introduce variables whose *P* values were smaller than 0.20 by univariate analysis. The following variables were entered into the model: age (year), pre-existing co-morbidity (Y/N), epileptic seizure (Y/N), status epilepticus (Y/N), GCS score at ICU admission, headache (Y/N), acute hypertension (Y/N), mean arterial pressure on scene (mmHg), highest glycaemia value (mmol/L), LOD score, grey matter involvement (Y/N), brainstem involvement (Y/N), ganglia involvement (Y/N), total number of brain areas involved, haemorrhagic complication at first imaging (Y/N), time from PRES onset to causative-factor control (hours), SAPS II score, duration of mechanical ventilation (days), mechanical ventilation (Y/N), refractory status epilepticus (Y/N), length of ICU stay (days), length of hospital stay (days), toxaemia of pregnancy (Y/N), and exposure to toxic agent (Y/N). Then, the absence of a significant increase in the likelihood value after omission of each of the remaining variables was checked. Variables were tested for co-linearity and interactions before inclusion in the multivariable model. Goodness of fit was evaluated by the Hosmer-Lemeshow statistic. The area under the ROC curve was estimated by the c statistic (Association of Predicted Probabilities and Observed Responses). Odds ratios (ORs) and their 95% confidence intervals (95%CIs) were computed. Values of *p* less than 0.05 were considered statistically significant. Analyses were done using the SAS 9.1 software package (SAS Institute, Cary, NC, USA).

**Table 7:** Multivariable analysis: Independent predictors of poor functional outcome on day 90.

	Odds Ratio	95%CI	*p* value
Toxaemia of pregnancy (preeclampsia/eclampsia)	0.06	0.01–0.38	**0.003**
Highest glycaemia (mmol/L) on day 1	1.22	1.02–1.45	**0.03**
Time from PRES onset to control of causative factor >30 min	3.30	1.04–10.46	**0.04**
Status epilepticus	1.66	0.52–5.29	0.39

Values of *p* in bold are significant (*p*<0.05).

Goodness of fit (Hosmer-Lemeshow) chi-square *p* value = 0.27.

Area under the ROC curve estimated by the c statistic = 0.31.

The following variables were entered into the model: age, pre-existing co-morbidity, epileptic seizure, status epilepticus, GCS score at ICU admission, headache, acute hypertension, mean arterial pressure on scene, highest glycaemia value, grey matter involvement, brainstem involvement, ganglia involvement, total number of brain areas involved, haemorrhagic complication at first imaging, time from PRES onset to causative-factor control (hours), SAPS II score, duration of mechanical ventilation, mechanical ventilation, refractory status epilepticus, length of ICU stay, length of hospital stay, toxaemia of pregnancy, and exposure to toxic agent.

95%CI, 95% confidence interval; PRES, posterior reversible encephalopathy syndrome.

## Results

### Patients (n = 70)

The patient flow chart is shown in Figure 1. Of the 86 patients admitted to the 24 study ICUs for possible PRES during the 9-year study period, 7 did not meet our inclusion criteria and 9 had no neuroimaging documents available for review, leaving 70 patients for the study.

Table 2 reports the main patient characteristics.

### On-scene Clinical Presentation (Figure 1)

Consciousness impairment was the most common clinical feature, being present in all but 4 (94%) patients. Clinical seizures occurred in 57 (81%) patients. Median value of mean arterial pressure was 122 (105–143) mmHg on scene. Acute hypertension was noted in 62 (89%) patients.

### Neuroimaging Features (Figure 1)

All patients underwent cerebral imaging. CT and MRI were performed in 12 (17%) and 58 (83%) patients, respectively. Among the 25 (36%) patients who underwent both CT and MRI, 4 (16%) had normal CT findings but had MRI evidence of PRES. Median time from ICU admission to brain imaging was 1 (0–4) day.

### Management

Median time from PRES onset to ICU admission was 0 (−2.5–0.5) hours. Mechanical ventilation was needed in 50 (71%) patients, for 5 (2–10) days. Median LOD score was 6 (IQR, 4–8), indicating a median of 3 (2–4) organ dysfunctions per patient. Organ involvements were as follows: neurologic, n = 54 (77%); respiratory, n = 50 (71%); renal, n = 44 (63%); haemodynamic, n = 22 (31%); hepatic, n = 12 (17%); and haematological, n = 15 (21%). Among patients with seizure activity, 31 (44%) had status epilepticus, including 7 (10%) refractory cases. EEG was performed in 59 (84%) patients and showed progression to electrical status epilepticus in 10 (17%). Overall, at least one cause of PRES was found in all 70 patients. The most common causes were toxicity (44%) and hypertensive encephalopathy (41%) (Table 3). Median time to control of the causative factor was 30 (7–86) hours. Systemic secondary brain insults on day 1 are reported in Table 4. Median lengths of ICU and hospital stays were 7 (IQR, 4–16) and 33 days (IQR, 15–62), respectively. ICU and hospital mortality rates were 10% (7 deaths) and 12.9% (9 deaths), respectively. During the 90-day follow-up period, 2 additional patients died, yielding an overall mortality rate of 16%. Among the 11 deaths, 4 were thought to be related to PRES, yielding a specific mortality rate of 5.7%. There was one case each of brain death related to cardiac arrest complicating the treatment of status epilepticus, refractory status epilepticus with multi-organ failure, cerebral haemorrhage with ventricular flooding, and brain death related to cerebral herniation complicating ischaemia. Among the 9 patients who died before hospital discharge, 6 died after decisions to forgo life-sustaining treatments.

### Ninety-day Functional Outcome

The day-90 GOS score was known for all 70 patients. Among them, 33 (47.1%) were alive with a GOS score of 5 (Figure 1). Independent predictors of day-90 functional outcome are reported in Table 7. Among them, two factors increased the risk of an unfavourable functional outcome, namely, higher maximal glycaemia on day 1 (OR, 1.22; 95%CI, 1.02–1.45, *P* = 0.03) and longer time to control of the causative factor (OR, 3.3; 95%CI, 1.04–10.46, *P* = 0.04). Toxaemia of pregnancy as the cause decreased the risk of an unfavourable outcome (OR, 0.06; 95%CI, 0.01–0.38, *p* = 0.003).

## Discussion

In this retrospective multicentre study of 70 patients with severe PRES, 11 (16%) patients died before day 90 and only 33 (56%) survivors had a good recovery defined as a GOS score of 5. Both of the factors independently associated with a poor outcome (GOS<5) were directly linked to the early management, and both may offer hope for improving outcomes (Panel).

Patient characteristics were consistent with previous studies. PRES preferentially affects women and relatively young individuals with serious co-morbidities. [9] The main clinical manifestations were consciousness impairment (94%) and seizure activity (81%). Consciousness was often severely impaired, with a median Glasgow Coma Scale score of 9 (3–14) at ICU admission. The 44% prevalence of status epilepticus was considerably higher than in previous reports of unselected cases of PRES. [1], [9] The other clinical signs such as headaches, vision impairment, nausea/vomiting, and focal neurological signs at scene were in accordance with recent reports. [1], [6], [8], [9], [29] Acute hypertension was found on-scene in 83% of patients, with a median value of mean arterial pressure level of 122 (105–143) mmHg, in keeping with earlier data. [1], [3], [8], [9] The neuroimaging findings in our study were also consistent with current knowledge. Despite a marked predominance of posterior parietal-occipital lobe involvement, atypical patterns were seen in a substantial proportion of patients and included strictly unilateral lesion distribution and involvement of the grey matter, anterior lobes, basal ganglia, cerebellum, and brainstem. Thorough familiarity with these atypical patterns is crucial, especially when the clinical features are not suggestive [6].

All patients received early symptomatic management concomitantly with prompt and extensive investigations to identify the cause. The on-scene presentation was severe as evidenced by the high number of organ failures, and mechanical ventilation was required in 71% of patients. Antihypertensive drugs and anticonvulsants were given early, as necessary. At least one cerebral imaging study was performed, and 83% of patients had MRI, the reference standard for diagnosing PRES. [30] At least one cause was identified in all patients, allowing early etiological treatment. Median time from PRES onset to control of the cause was only 30 (7–86) hours. Exposure to toxic agents was the most common cause, with 44% of patients. The already long list of toxic agents associated with PRES is growing steadily. In our study, immunosuppressants such as anticalcineurin agents (cyclosporine A and tacrolimus FK506) were the most common toxic causes. Hypertensive encephalopathy was the second most common causative factor, with 41% of patients. Toxaemia of pregnancy and autoimmune diseases were identified in 23% and 11% of patients, respectively, potentially explaining the female predominance of the syndrome. Finally, other factors reported in association with PRES, sometimes only anecdotally, include hypomagnesaemia, sickle cell disease, and infection/sepsis/shock.

We identified three factors independently associated with the day-90 functional outcome. Among them, time to control of the causative factor provides the greatest room for improvement. Although a role for this variable was suggested previously [11], [31], our study is the first to demonstrate an independent association with the outcome. Patients may require blood pressure control, withdrawal of cancer chemotherapy or immunosuppressants, caesarean section, dialysis, or other interventions. Prompt control of the cause is crucial to decrease the risk of ischaemia or bleeding, thereby avoiding permanent disabilities or death. [1] Although control of the causative factor was achieved within a median of only 30 hours in our study, ischaemic and haemorrhagic complications occurred in 8% and 12% of patients, respectively. Moreover, reversibility of the imaging abnormalities was partial in 43% and complete in 45% of patients. Several studies have suggest that toxaemia of pregnancy may be associated with better outcomes. [32] In our study, toxaemia of pregnancy was independently associated with a higher likelihood of a favourable day-90 outcome (GOS = 5). Maternal mortality after eclampsia is only about 1%. However, extra-neurological complications (placental abruption and HELLP syndrome) and neurological complications (ischaemia and haemorrhage) can occur. Another risk after eclampsia is the development of post**-**traumatic stress disorder. Hyperglycaemia was the last factor independently associated with the 90-day outcome in our patients. Hyperglycaemia, a common occurrence in neurocritical care patients [33], was significantly associated in several studies with increased mortality and impaired functional outcomes after events such as stroke [34], [35], intracerebral haemorrhage [36], traumatic brain injury, [37] and spinal cord injury. [38] However, whether hyperglycaemia is an independent prognostic factor or a marker for brain injury severity remains unclear. [33].

None of the clinical or neuroimaging features evaluated in our study was independently associated with the day-90 outcome. The two independent risk factors for a poor day-90 outcome were related to early management. Our results support a goal-directed strategy combining prompt symptomatic treatment and control of the causative factor as soon as it is identified, regardless of on-scene severity of the clinicoradiologic presentation.

Previous studies provided limited data on the management and outcome of PRES. The mortality rate reached 15% [8], [9]. An assessment of functional impairment was reported in a single study, which found a median modified Rankin scale of 2.5 at discharge, indicating mild-to-moderate disability [8]. None of these studies focused on severe PRES. Moreover, the relative roles in fatal outcomes of the syndrome itself and of associated factors remained unclear. Our multicentre study accurately delineates the presentation, management, and determinants of functional outcomes in patients requiring ICU admission for severe forms of PRES.

Our study has several limitations. First, the extent to which our findings apply to the full spectrum of patients with PRES is unclear. Patients were included in 24 ICUs over a 10-year period, yielding a great variability in the radiologic technology used for diagnosis and follow-up. However the year of admission, as well as the center, were not determinants of functional outcome. However, despite the retrospective study design, the participation of 24 ICUs provided a broad picture of the management of severe PRES. Second, PRES was diagnosed based on a combination of clinical and neuroimaging features previously described as consistent with PRES. MRI was obtained in most of the patients, all neuroimaging studies were reviewed by two certified senior neurologists blinded to clinical data, and patients were included by consensus between the two neurologists. Consensus agreement between the two neurologists that performed independent blinded review of all available neuroimaging was strong with a value of 1 (considering a proportion of concordant classifications expected by chance of 0.05). Given the 7 cases discarded after neuroimaging expertise, kappa coefficient agreement between initial se*le*ction of cases and final enrolment was excellent with a value of 0.9 (considering a proportion of concordant classifications expected by chance of 0.05). Follow-up neuroimaging studies were lacking in 30% of patients, but this proportion was even higher in previous reports [6], [8]. Third, the GOS in its structured form has been found valid, practical, and reliable [39], but indirect GOS evaluation via charts or physician interviews has not been studied. Qualitative data from structured interviews may better assess the cognitive and emotional burden after PRES. Fourth, A GOS score of 4 may be viewed as a favorable outcome. A GOS score of 4 is “moderate disability” in a “patient capable of living independently but unable to return to work or school”. This category is associated with functional impairments, as “some previous activities, either at work or in social life, are now no longer possible by reason of either physical or mental deficit”. Thus, in keeping with other studies of outcomes of neurological conditions in critically ill patients, we defined a poor outcome as a GOS score smaller than 5. [40], [41] Fifth, even if global mortality rate was of 15.7%, assessment of direct imputability of PRES indicates a lower value of 5.7%. This interesting finding reinforces results of our multivariate analysis of factors associated with functional outcome in patients with severe PRES. Indeed, toxemia of pregnancy, as opposed to others causes, was associated with good outcome, underlying burden of co-morbidities in this syndrome. Moreover, reasons for death directly ascribable to PRES demonstrate the potential of aggravation in course of disease of patients presenting with severe PRES. Sixth, impact on 90-days functional outcome of other organ dysfunction than respiratory failure was not assessed during the whole ICU stay. Seventh, due to the retrospective design of the study, the GOS score was extracted from the hospital charts or determined by interviewing the patient’s general practitioner or neurologist in 90% of patients. In the remaining 10% of patients (corresponding to latest inclusions), the GOS score was evaluated by the local investigator, who administered the scale directly to the patient during a phone interview. The day-90 GOS score was known for all study patients whereas the day-180 GOS score was known for 62 (88.6%) patients. Last, 90 days after ICU discharge may be too early to determine the final functional outcome. However, 90 days is the most widely used interval for assessing overall outcomes after brain injuries.

### Conclusions

In conclusion, 90 days after severe PRES only 56% of survivors had achieved a good recovery and the outcome was predicted chiefly by factors available within a few hours after ICU admission. Among these factors, hyperglycaemia during the first 24 hours and delayed control of the cause may be amenable to improvement. These results should be confirmed in a large multicentre prospective study.
