# Correction: Determinants of Recovery from Severe Posterior Reversible Encephalopathy Syndrome

**DOI:** 10.1371/annotation/2d87c752-042a-4c61-9254-9a3c73620bcd

**Published:** 2013-11-01

**Authors:** Stephane Legriel, Olivier Schraub, Elie Azoulay, Philippe Hantson, Eric Magalhaes, Isaline Coquet, Cedric Bretonniere, Olivier Gilhodes, Nadia Anguel, Bruno Megarbane, Laurent Benayoun, David Schnell, Gaetan Plantefeve, Julien Charpentier, Laurent Argaud, Bruno Mourvillier, Arnaud Galbois, Ludivine Chalumeau-Lemoine, Michel Rivoal, François Durand, Arnaud Geffroy, Marc Simon, Annabelle Stoclin, Jean-Louis Pallot, Charlotte Arbelot, Martine Nyunga, Olivier Lesieur, Gilles Troché, Fabrice Bruneel, Yves-Sébastien Cordoliani, Jean-Pierre Bedos, Fernando Pico

There is an error in the time listed in third row of Table 7. The correct sentence should read “Time from PRES onset to control of causative factor > 30 hours.” 

